# Comparison of hepatic resection and transarterial chemoembolization for UICC stage T3 hepatocellular carcinoma: a propensity score matching study

**DOI:** 10.1186/s12885-018-4557-5

**Published:** 2018-06-07

**Authors:** Chong Zhong, Yong-Fa Zhang, Jun-Hai Huang, Cheng-Ming Xiong, Zi-Yu Wang, Qing-Lian Chen, Rong-Ping Guo

**Affiliations:** 1 Lingnan Medical Research Center, Guangzhou University of Chinese Medicine, 16 Airport Road, Guangzhou, 510405 China; 2 Department of Hepatobiliary Surgery, the First Affiliated Hospital of Guangzhou University of Chinese Medicine, 16 Airport Road, Guangzhou, 510405 China; 3 Department of Liver Surgery, Fudan University Shanghai Cancer Center, Shanghai, 200032 China; 4 Department of Oncology, Shanghai Medical College, Fudan University, Shanghai, 200032 China; 5 The First Clinical Medical School of Guangzhou University of Chinese Medicine, Guangzhou, 510405 China; 6 Department of Hepatobiliary Oncology, Cancer Center of Sun Yat-sen University, Guangzhou, 510060 China

**Keywords:** Hepatocellular carcinoma, Hepatic resection, TACE, Propensity score matching study

## Abstract

**Background:**

The optimal therapeutic strategy in UICC stage T3 hepatocellular carcinoma (HCC) patients that maximizes both safety and long-term outcome has not yet been determined. Our aim was to compare clinical outcomes following hepatic resection (HR) versus transarterial chemoembolization (TACE) for stage T3 HCC.

**Methods:**

From 2005 to 2013, 1179 patients with T3 HCC who underwent HR or TACE were divided into two groups, HR group (*n* = 280) or TACE group (*n* = 899). The clinical outcomes were compared before and after propensity score matching.

**Results:**

The propensity model matched 244 patients in each group for further analyses. After matching, medium overall survival (OS), 1, 3, and 5-year OS rates in TACE group were 11.8 (95%CI, 9.9–13.7) months, 49.6, 16.5, and 8.4%, respectively; which in HR group were 17.8 (95% CI, 14.8–20.8) months, 63.1, 33.3, and 26.4%, respectively; (log rank = 19.908, *P* < 0.01). Patients in HR group were more likely to develop pleural effusion, compared with those in TACE group (0.4% vs. 5.3%, *P* = 0.01). However, no significant differences in other adverse events (AEs) were found between two groups. Similar results were also demonstrated prior to the matched analysis. Multivariate analysis indicated that prothrombin time (PT), tumor size, tumor numbers, UICC staging status, and initial treatment were independent prognostic factors.

**Conclusions:**

Our study revealed that TACE was an option for UICC T3 HCC patients. However, HR seemed to be safe and yield a survival benefit compared with TACE, especially for patients with a good underlying liver function.

## Background

Hepatocellular carcinoma (HCC) has been the second leading cause of cancer death worldwide so far, estimated to be responsible for around 9.1% of the total cancer death [1]. It is the only cancer that mortality is still increasing regardless of the evolution and progress of anti-cancer therapy in North America [2]. As in China, more than 50% new developed cases occurred in this country alone, which usually arises as a result of a chronic liver disease, especially hepatitis B virus (HBV) related. Due to its greatly invasive malignant features, HCC has a characteristic propensity to invade into portal vein, or to develop intra-hepatic metastasis, which was regarded as one of the most adverse prognostic factors [3]. Although several staging systems have been proposed for determining the stage and prognosis of HCC, no consensus exists on the best classification system [2, 4]. Until now the Union for International Cancer Control and American Joint Committee on Cancer (UICC/AJCC) tumor-node-metastasis (TNM) staging system has still served as one of the most important staging systems all over the world [4].

UICC stage (7th) T3 HCC was defined as multiple lesions with any lesion larger than 5 cm (stage IIIa), or involving a major portal vein or hepatic veins (stage IIIb). According to Barcelona clinic liver cancer (BCLC) staging system, most UICC stage T3 HCC cases are classified as being Stages B or C, therefore transarterial chemoembolization (TACE) or Sorafenib, rather than hepatic resection (HR) are recommended as the optimal therapy for patients in these stages in Europe or North America [5]. However, therapy strategy may be a little different in Asian-Pacific areas [2, 3, 6, 7]. Until now, it seems difficult to reach a common consensus on the indication of HR for HCC patients worldwide. Actually, HR was reported to be performed beyond the BCLC recommendations in about 50% of HCC patients with BCLC stage B or C in Asia-Pacific areas [6, 8, 9]. It’s not yet clear what is the optimal therapeutic strategy for UICC stage T3 HCC patients. The aim of this study was to compare the clinical outcomes following HR versus TACE for UICC stage T3 HCC.

As we known, the underlying liver cirrhosis and tumor characteristics make a significant contribution to the prognosis of HCC patients. Without balancing the biases of liver cirrhosis and tumor characteristics can cause confusion. Therefore, non-randomized studies that compare the outcomes of HR and TACE in HCC patients without balancing the biases should be interpreted cautiously. Methods of balancing co-variables are needed in this specific setting. In nonrandomized studies, propensity score matching (PSM) is an optimal method of reducing the biases of treatment selection [10]. Compared with the traditional adjustment methods (stratification and covariance adjustment), PSM maximizes the covariate balance between groups and is free of the limitations of adjusting a limited number of covariates at one time [11]. Therefore, in the present study, we conducted PSM to minimize the biases to assess long-term outcomes of HR versus TACE for UICC stage T3 HCC.

## Methods

### Patients

**Fig. 1:**
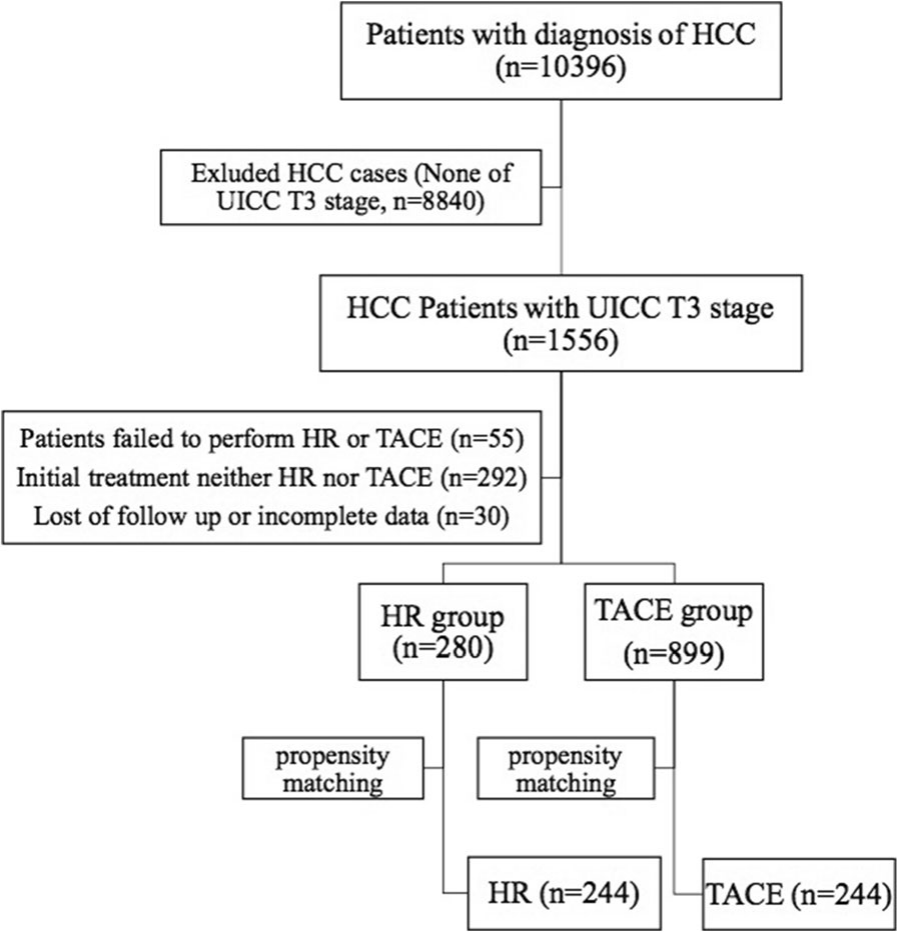
Flow chart of the study and the treatment strategies of patients with UICC stage T3 HCC

The study protocol was approved by the Ethics Committee of Sun Yat-Sen University Cancer Center and The First Affiliated Hospital of Guangzhou University of Chinese Medicine. All recruited patients provided written informed consent before HR or TACE. Between January 2005 and December 2013, 10,396 consecutive patients with the diagnosis of HCC at the Department of Hepatobiliary Oncology at Sun Yat-Sen University Cancer Center and the Department of Hepatobiliary Surgery at the First Affiliated Hospital of Guangzhou University of Chinese Medicine were considered amenable for this study. Of these, 1179 patients met the inclusion criteria defined as that in the our previous studies [3, 12]: (1) a confirmed diagnosis of HCC with no previous treatment; (2) Chronic liver disease with compensated cirrhosis (Child-Pugh grade, A or B) or without underlying chronic liver disease; (3) multiple tumors with at least one lesion more than 5 cm or tumor involving a major branch of the portal vein; (4) tumor lesions suit for potentially radical hepatic resection with a negative resection margin. As we described in the previous articles [3, 12]. Briefly, the criteria of potentially radical hepatic resection in our study are as follows, the tumor with multiple lesions localized in right or left hemi-liver, or the main tumor localized in one lobe only with a small solitary lesion in contralateral lobe, or tumor involving a major branch (the first or second branch) of the portal or hepatic vein (s), which could be safely resected without grossly remaining tumors. That was regarded as potentially radical hepatic resection. At the same time, a well preserved postoperative liver function of patients was anticipated, which was assessed by our surgical team according to the criteria defined in the other previous article that remnant liver volume no less than 250 ml/m^2^ [13] (Fig. 1).

The criteria of exclusion were as follows: (1) patients that failed to perform hepatic resection or TACE, such as serious concurrent medical illness, or platelet count (PLT) less than 50 × 10^9^/L, or Child–Pugh grade C, et al.; (2) other therapies, rather than HR or TACE, used as the initial treatment; (3) lack of follow up or incomplete data.

### Strategies for hepatic resection and TACE

Hepatic resection strategy was defined as potentially radical resection, detailed in our previous reports [3, 12, 14]. Briefly, intraoperative ultrasonography was performed routinely to assess the numbers and size of tumor lesions and the relationship between tumors and vessels. Pringle’s maneuver was routinely used with a switch of clamp and unclamp time of 10 min and 5 min. Anatomic resection was our preferred surgical method for multiple nodules. For multiple bi-lobar nodules, anatomic resection was conducted for the main tumor, whereas satellite nodules were non-anatomically resected with a negative resection margin. In order to preserve adequate post-operative liver function, non-anatomic resection was performed with a negative resection margin for some specific cases. The negative resection margin was defined as in our previous reports [3, 12, 14]. The en bloc technique was our preferable technique in the surgical management for the patients with portal vein invasion [3]. TACE was carried out using the same drug regimens and techniques that we described previously [12, 15], and TACE was performed by four radiologists who each had 7–10 years of experience with TACE.

### Propensity score analysis

We conducted PSM to minimize the bias that arises from patient backgrounds to assess the safety and long-term outcome of HR versus TACE for UICC stage T3 HCC. Possible variables associated with clinical characteristics of HCC patients, including age, gender, etiology, serum biochemistries, Child-Pugh (C-P) grade, albumin–bilirubin (ALBI) level, tumor size, tumor numbers, and UICC stage were comprehensively selected for one-to-one propensity score matching analysis.

### Follow-up

Complications were defined as complications within the 90 days after treatment. Common Terminology Criteria for Adverse Events V3.0 were used to grade the severity of adverse events and complications [16].

The time to progression (TTP), according to the National Cancer Institute (NCI) dictionary of cancer terms, was defined as the length of time from the date of diagnosis or the start of treatment for a disease until the disease starts to get worse or spread to other parts of the body. We just used TTP to see how well TACE worked in TACE group. But it was hard to used it in HR group. The duration of follow-up was defined as the interval between the date of HR or TACE, and the date of death or the last time of follow-up. Data in this study were censored on December 31, 2016. All patients were followed up at an interval of 2–3 months during the first 2 years after initial therapy, then 3–6 months after 2 years. The Strategies of follow-up involved physical examination, serum alpha-fetoprotein (AFP), abdominal color ultrasonography, and chest X-ray (optional). Computer tomography (CT), magnetic resonance imaging (MRI), and/or hepatic angiography were conducted upon suspicion of recurrence and/or metastasis. If necessary, biopsy under guidance of ultrasonography or CT was performed to confirm the diagnosis. The diagnosis of tumor relapse or metastasis was based on the criteria for HCC used by the American Association for the Study of Liver Diseases (AASLD) [2]. The numbers and the location of recurrent and/or metastatic HCC were recorded when the diagnosis was established. The recommended therapy strategies from our multidisciplinary team [3, 14], involving potentially radial therapies, such as hepatic resection, radiofrequency ablation, microwave thermotherapy, even liver transplantation; or loco-regional therapy such as TACE, or Sorafenib, or systemic therapy for those recurrent or metastatic cases were determined by the characteristics of tumor lesions, performance status (PS), liver function of the patients. Conservative treatments were provided for patients with terminal HCC, liver function of Child-Pugh grade C, or PS scores > 2.

### Statistical analysis

SPSS 21.0 (IBM, New York, NY) software was applied to analyze the data. Measurement data were expressed as means ± standard deviations (SDs), and comparisons among groups were analyzed by analysis of variance (ANOVA) or t tests. Enumeration data were expressed as rates, and comparisons among groups were analyzed by chi-square tests. Matched package was used to produce the propensity score graphs. Co-variables entered into the model included age, gender, etiology, liver function (including PT, ALB, TBL, C-P grade and ALBI grade), tumor burden (AFP, tumor size, tumor numbers, UICC stage) [17]. One-to-one match between HR group and TACE group was obtained by use of the nearest neighbor matching. In addition, a penalty was added when the propensity scores differed by more than 0.2 times the SD. Survival curves were generated using the Kaplan-Meier method with the log-rank test. Univariate and multivariate analyses of overall survival using stepwise variable selection procedure of Cox regression model was assessed. Differences with 2-sided *P* values of less than 0.05 were considered statistically significant.

## Results

### Baseline of patient characteristics before and after PSM

**Fig. 2:**
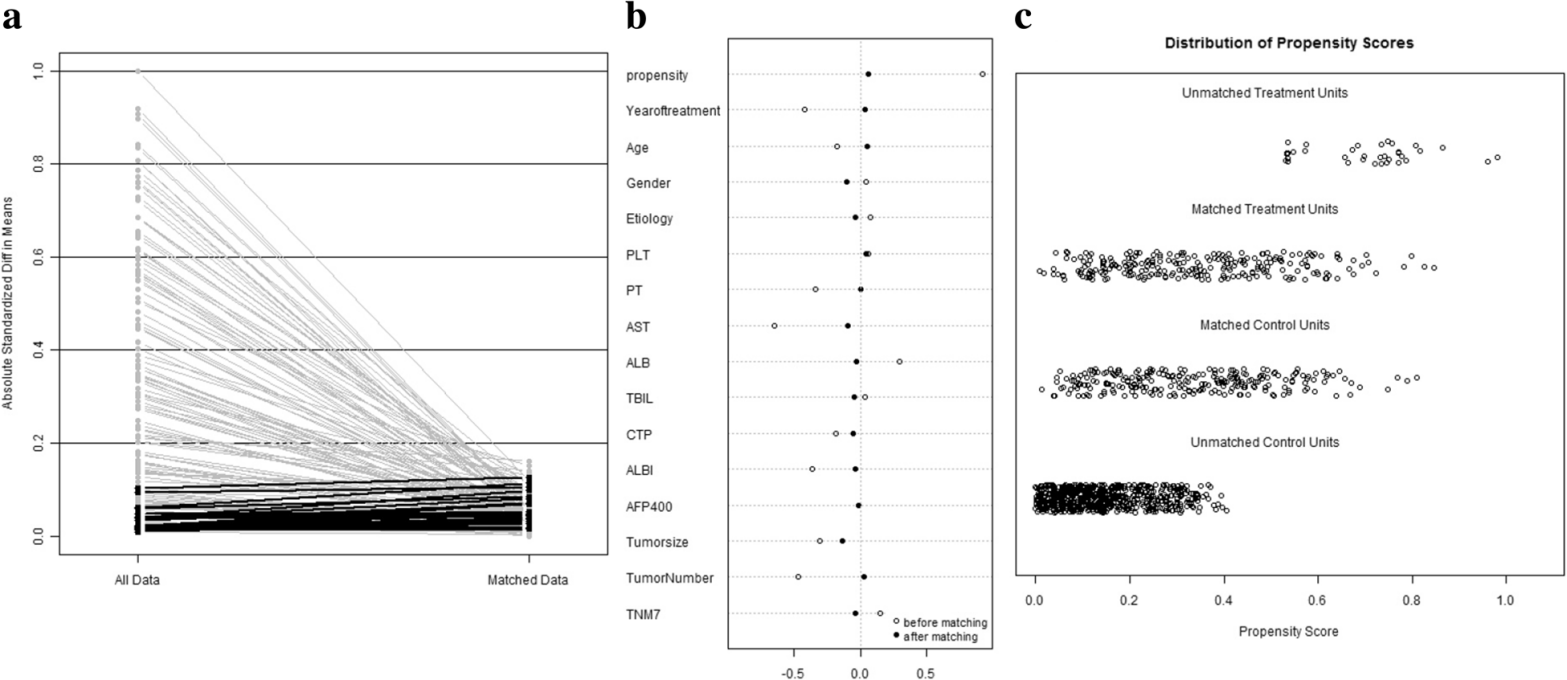
Line plots of standardized differences of this study before and after propensity score matching. A: Parallel line plot of the standardized difference in means before and after PSM; B and C: Dot plot of the propensity scores of patients in HR and TACE group

We compared the baseline characteristics of patients who received TACE (*n* = 899) and HR (*n* = 280) in Table 1. The most frequent etiology was chronic hepatitis B virus (HBV) in both the TACE and HR groups (90% vs. 88%, *P* = 0.216). Compared with patients in the year from 2005 to 2009, more patients in the year from 2010 to 2014 received TACE (60% vs. 40%, *P* < 0.001). Compared with patients in HR group, patients in the TACE group revealed older (51.0 vs. 48.8 years, *P* = 0.009), larger tumors (9.5 cm vs. 8.5 cm, *P* < 0.001), more cases of more than one lesion (81% vs. 57%, *P* < 0.001), more patients of UICC stage IIIa (56% vs. 49%, *P* = 0.033). In particular, patients in the TACE group have longer prothrombin time (PT) (12.7 vs. 12.3 s, *P* < 0.001), higher aspartate aminotransferase (AST) level (80.7 vs. 55.4 U/L, *P* < 0.001), lower serum albumin (ALB) levels (40.0 vs. 41.1 g/l, *P* < 0.001), less cases of C-P grade A (95% vs. 98%, *P* = 0.049) and less cases of Albumin-Bilirubin (ALBI) grade 1 (52% vs. 69%, *P* < 0.001). We conducted PSM analysis to minimize the bias according to the methods recommended by D’Agostino [10]. After matching, 488 patients (each group 244 patients) were matched and selected for further analyses (Fig. 2). After matching, there were no significant differences between the TACE and HR groups (Table 1).

**Table 1:** Demographics and clinical characteristics of HCC patients before and after one-to-one propensity score matching analysis

Variables	All patients			Propensity-matched patients		
	HR (*n* = 280)	TACE (*n* = 899)	*P* value	HR (*n* = 244)	TACE (*n* = 244)	*P* value
Year of treatment (−09/10-) [n (%)]	169(60)/111(40)	359(40)/540(60)	< 0.001	137(56)/107(44)	141(58)/103(42)	0.715
Age (y)	48.77 ± 12.90	51.04 ± 11.47	0.009	49.5 ± 13.0	48.9 ± 11.9	0.584
Gender (male/female) [n (%)]	254(91)/26(9)	826(92)/73(8)	0.539	222(91)/22 (9)	215(88)/29 (12)	0.300
Etiology (HBV related/none) [n (%)]	245 (88)/35(12)	810 (90)/89 (10)	0.216	212 (87)/ 32(13)	209 (86)/35(14)	0.693
PLT (10^9^/L)	204.9 ± 81.9	199.8 ± 88.8	0.395	200.9 ± 80.0	197.4 ± 79.0	0.622
PT (sec)	12.3 ± 1.2	12.7 ± 1.4	< 0.001	12.3 ± 1.2	12.3 ± 1.3	0.955
AST (U/L)	55.4 ± 39.5	80.7 ± 65.3	< 0.001	56.7 ± 41.2	60.3 ± 30.9	0.271
ALB (g/L)	41.1 ± 4.0	40.0 ± 4.2	< 0.001	41.0 ± 3.8	41.1 ± 4.0	0.769
TBIL (μmol/L)	19.8 ± 40.4	18.2 ± 12.1	0.525	16.1 ± 18.3	17.8 ± 17.9	0.298
C-P grade (A/B) [n (%)]	273(98)/7(2)	851(95)/48(5)	0.049	240(98)/4 (2)	238(98)/6 (2)	0.523
ALBI (level 1/level 2–3) [n (%)]	193(69)/87(31)	469(52)/430(48)	< 0.001	165(68)/79 (32)	161(66)/83(34)	0.701
AFP (≤ 400 μg/L / > 400 μg/L) [n (%)]	127(45)/153(55)	402(45)/497(55)	0.851	113(46)/131(54)	115(47)/129 (53)	0.856
Tumor size (cm)	8.5 ± 3.2	9.5 ± 3.2	< 0.001	9.0 ± 3.1	8.6 ± 3.2	0.147
Tumor numbers (1/2-) [n (%)]	119(43)/161(57)	175(19)/724(81)	< 0.001	88/156 (36/64)	85/159 (35/65)	0.776
UICC stage (IIIa / IIIb) [n (%)]	136(49)/144(51)	502(56)/397(44)	0.033	129(53)/115(47)	134(55)/110(45)	0.650

Variables are expressed as mean ± SD or no. (%), unless otherwise indicated

*Abbreviations*: *TACE* transarterial chemoembolization, *HR* hepatic resection, *HBV* hepatitis B virus, *PLT* platelet count, *PT* prothrombin time, *AST* aspartate aminotransferase, *ALB* albumin, *TBIL* total bilirubin, *C-P* Child-Pugh, *ALBI* albumin–bilirubin, *AFP* alpha-fetoprotein, *UICC* the Union for International Cancer Control

### Outcome and overall survival

Before matching, the median follow-up period was 36.8 (range, 1.1–137.1) months for the HR group and 25.7 (range, 0.9–134.4) months for the TACE group. Before matching, the median procedures of TACE were (1.8 ± 1.2) procedures. 457 (50.8%) cases just took 1 procedure of TACE, 264 (29.4%) and 101 (11.2%) cases took 2 and 3 procedures of TACE, respectively. 77 (8.6%) cases took more than 3 procedures of TACE. 419 (46.6%) cases developed progress after initial TACE. In these cases, 186 (20.7%) cases developed lesion enlarging or new lesion occurred. 233 (25.9%) cases developed distant metastasis or vessel invasion or vessel invasion progressed. The median TTP was 5.7 [95% confidence interval (CI), (4.7–6.6)] months. There were 135 (15.0%), 93 (10.3%), and 86 (9.6%) cases received heat ablation, resection, and Sorafenib treatment after the initial treatment of TACE, respectively (Table 2).

**Table 2:** Outcome of TACE before and after propensity score matching analysis in UICC T3 HCC patients

Variables	Before PSM	After PSM
	TACE (*n* = 899)	TACE (*n* = 244)
Procedures (mean ± SD)	1.8 ± 1.2	1.9 ± 1.2
1 procedure	457 (50.8)	118(48.3)
2–3 procedures	365 (40.6)	103(42.2)
more than 3 procedures	77 (8.6)	23(9.4)
Best tumor response, n (%)		
CR	18 (2.0)	6 (2.5)
PR	203 (22.6)	56 (23.0)
SD	259 (28.8)	73 (29.9)
DCR (CR + PR + SD)	480 (53.4)	135 (55.3)
Median TTP (95%CI) months	5.7(4.7–6.6)	7.7 (5.7–9.8)
Cases of PD, n (%)	419 (46.6)	109 (44.7)
Patterns of PD		
Enlarged or new lesion (n, %)	186 (20.7)	49 (20.1)
Metastasis or vessel invasion (n, %)	233 (25.9)	60 (24.6)
Treatment after TACE		
Heat ablation (n, %)	135 (15.0)	43 (17.6)
Resection (n, %)	93 (10.3)	27 (11.1)
Sorafenib (n, %)	86 (9.6)	21 (8.6)

*Abbreviations*: *UICC* the Union for International Cancer Control, *TACE* transarterial chemoembolization, *CR* complete response, *PR* partial response, *SD* stable disease, *DCR* disease control rate, *TTP* time to progression, *CI* confidence interval, *PSM* propensity score matching

**Fig. 3:**
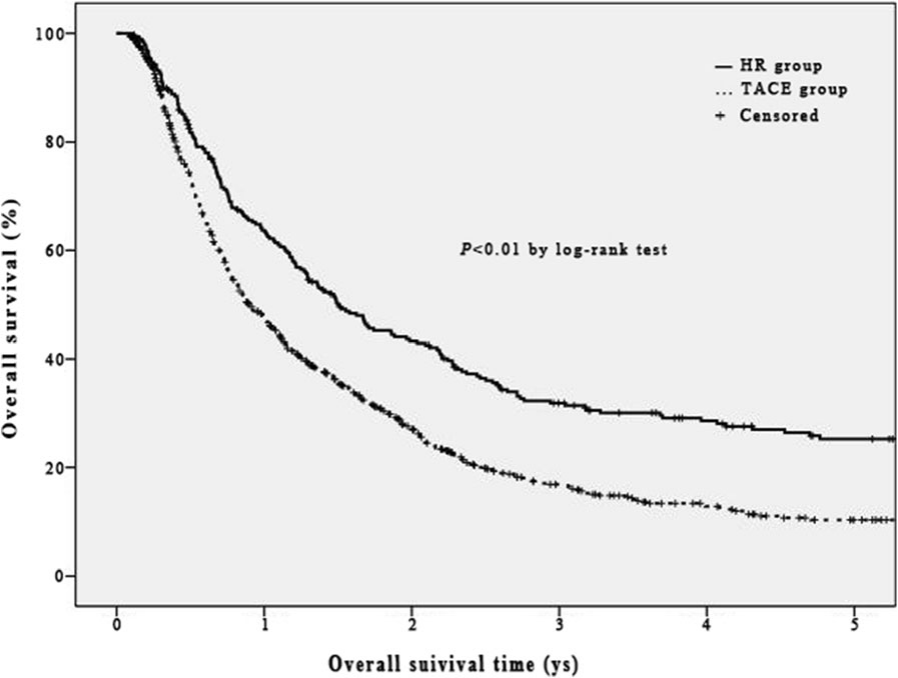
Overall survival curves of UICC T3 HCC patients in HR group and TACE group before propensity score matching

The medium overall survival (OS), 1, 3, and 5-year OS rates were 10.9 (95% CI, 9.7–12.1) months, 47.1, 16.9, and 10.3%, respectively, which were lower than those in HR group significantly [18.0 (95% CI, 14.1–21.9) months, 63.7, 31.9, and 25.3%; log rank = 32.979, *P* < 0.01, Fig. 3].

**Fig. 4:**
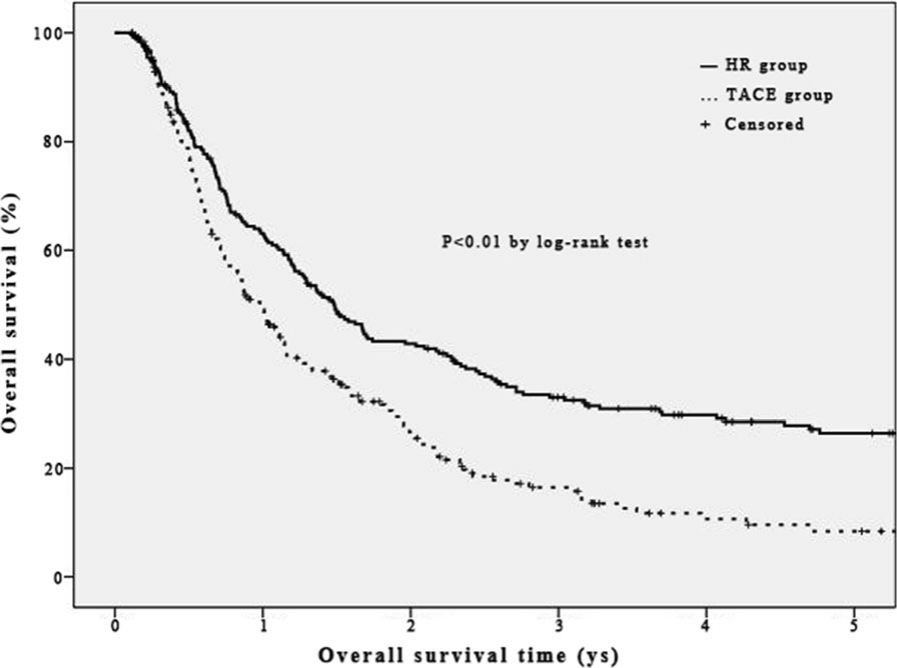
Overall survival curves of UICC T3 HCC patients in HR group and TACE group after propensity score matching

After matching, the mean procedures of TACE were (1.9 ± 1.2) procedures. Median TTP 7.7 (95% CI, 5.7–9.8) months. 49 (20.1%) cases developed enlarged or new lesion in liver, whereas 60 (24.6%) cases developed metastasis or vessels invasion. There were 43 (17.6%), 27 (11.1%), and 21 (8.6%) cases performed heat ablation, resection, and Sorafenib after initial TACE, respectively (Table 2). The medium OS, 1, 3, and 5-year OS rates in TACE group were 11.8 (95% CI, 9.9–13.7) months, 49.6, 16.5, and 8.4%, respectively, which were lower than those in HR group significantly [17.8 (95% CI, 14.8–20.8) months, 63.1, 33.3, and 26.4%; log rank = 19.908, *P* < 0.01, Fig. 4].

### Safety and mortality

Adverse events (AEs) related to TACE and HR within 90 days after treatment are shown in Table 3. Before matching, patients in the TACE group have less cases to develop grade 3–4 edema (0.2% vs. 0.7%, *P* = 0.031), grade 3–4 of fever (1.8% vs. 4.6%, *P* < 0.01), grade 3–4 of ascites (0.3% vs. 1.8%, *P* = 0.03), and pleural effusion (0.2% vs. 5.7%, *P* < 0.01), respectively. No significant differences in other AEs were found between two groups, including grade 3–4 of pain, vomiting, upper gastrointestinal hemorrhage (UGIH), postoperative hemorrhage (POH), renal failure, liver dysfunction, and bile leakage. 2 and 3 cases developed treatment-related deaths (TRD) in TACE and HR group, respectively (*P* = 0.09). After matching, patients in HR group were more likely to develop pleural effusion, compared with those of TACE group (0.4% vs. 5.3%, *P* = 0.01). However, no significant differences in other AEs were found between two groups.

**Table 3:** Postoperative adverse events before and after propensity score matching analysis in UICC T3 HCC patients

Variables	Before PSM			After PSM		
	TACE (*n* = 899) HR (*n* = 280) *P* value			TACE (*n* = 244) HR (*n* = 244) *P* value		
Pain (0–2/3–4)	899/1	278/2	0.142*	243/1	243/1	1.0
Edema (0–2/3–4)	899/2	277/3	0.031*	243/1	242/2	1.0*
Fever (0–2/3–4)	883/16	267/13	0.007	241/3	240/4	1.0*
Vomiting (0–2/3–4)	888/11	278/2	0.70^#^	238/6	242/2	0.285*
Ascites (0–2/3–4)	896/3	275/5	0.03^#^	242/2	240/4	0.686*
Pleural effusion (0–2/3–4)	897/2	264/16	< 0.01^#^	243/1	231/13	0.01
UGIH/POH (0–2/3–4)	897/2	278/2	0.241*	243/1	242/2	1.0*
Renal failure (0/1–2)	896/3	279/1	1.0*	243/1	242/2	1.0*
Liver dysfunction (0–2/3–4)	898/1	278/2	0.142*	243/1	243/1	1.0
Bile leakage (n/y)	898/1	279/1	0.422*	244/1	243/1	1.0*
TRD	897/2	277/3	0.090*	243/1	243/1	1.0*

*Abbreviations*: *UICC* the Union for International Cancer Control, *UGIH* upper gastrointestinal hemorrhage, *POH* postoperative hemorrhage, *TRD* treatment-related death, *PSM* propensity score matching

* Fisher’ exact test. ^#^ χ^2^ test with a continuity correction

### Univariate and multivariate analyses of overall survival for patients before and after the PSM analysis

To investigate the impacts of patient demographics and clinical characteristics on the outcomes of OS, the variables listed in Table 1 were included in the univariate and multivariate analysis. Before matching, in the multivariate analysis, the prothrombin time (PT) (hazard ratio, HR = 1.167; 95%CI, 1.002–1.359; *P* = 0.047), AST (aspartate aminotransferase) (HR = 1.232; 95% CI, 1.055–1.439; *P* = 0.008), ALBI (HR = 1.246; 95% CI, 1.084–1.431; *P* = 0.002), tumor size (HR = 1.235; 95% CI, 1.071–1.424; *P* = 0.004), tumor numbers (HR = 1.334; 95% CI, 1.098–1.620; *P* = 0.004), UICC stage (HR = 1.831; 95% CI, 1.545–2.171; *P* < 0.001), year of treatment (HR = 0.869; 95% CI, 0.746–0.993; *P* = 0.039), and initial treatment (HR = 0.677; 95% CI, 0.569–0.806; *P* < 0.001) were identified as independent predictors of OS (Table 4).

**Table 4:** Univariate and multivariate analyses of overall survival for patients before and after propensity score matching analysis in UICC T3 HCC patients

Variable	OS before PSM				OS after PSM			
	Univariate analysis	Multivariate analysis			Univariate analysis	Multivariate analysis		
	P	P	HR	95%CI	P	P	HR	95%CI
Age (y), ≥/< 60	0.613				0.131			
Gender (female/male)	0.325				0.776			
Etiology (others/HBV)	0.161				0.296			
PLT (109/L), ≥/< 100	0.994				0.356			
PT (sec), ≥/< 13	0.069	0.047	1.167	1.002–1.359	0.087	0.003	1.425	1.128–1.800
AST (U/L), >/≤ 45	0.019	0.008	1.232	1.055–1.439	0.304			
ALB (g/L), ≥/< 35	0.403				0.058			
TBL (mmol/L), >/≤ 17	0.408				0.937			
(C-P) grade A/B, C	0.400				0.338			
ALBI (grade2–3/ grade 1)	0.027	0.002	1.246	1.084–1.431	0.810			
AFP (ng/mL), ≥/< 400	0.119				0.291			
Tumor size (cm) ≥/< 10	0.016	0.004	1.235	1.071–1.424	0.030	0.003	1.406	1.125–1.757
Tumor numbers (n), > 1/1	0.004	0.004	1.334	1.098–1.620	0.022	0.042	1.435	1.014–2.030
UICC stage IIIb /IIIa	0.000	0.000	1.831	1.545–2.171	0.000	0.000	1.831	1.311–2.559
Year of treatment (10−/−09)	0.056	0.039	0.860	0.746–0.993	0.163			
Initial treatment (HR/TACE)	0.000	0.000	0.677	0.569–0.806	0.000	0.000	0.646	0.522–0.798

*Abbreviations*: *PLT* platelet count, *PT* prothrombin time, *AST* aspartate aminotransferase, *ALB* albumin, *TBL* total bilirubin, *(C-P) grade* child-Pugh grade, *ALBI* albumin-bilirubin grade, *AFP* alpha-fetoprotein, *UICC* the Union for International Cancer Control, *HR* hepatic resection, *TACE* transcarterial chemoembolization, *PSM* propensity score matching

PT (HR = 1.425; 95% CI, 1.128–1.800; *P* = 0.003), tumor size (HR = 1.406; 95% CI, 1.125–1.757; *P* = 0.003), tumor numbers (HR = 1.435; 95% CI, 1.014–2.030; *P* = 0.042), UICC stage (HR = 1.831; 95% CI, 1.311–2.559; *P* < 0.001), and initial treatment (HR = 0.646; 95% CI, 0.522–0.798; *P* < 0.001) were identified as independent predictors of OS after matching as shown by the multivariate analysis.

## Discussion

HCC is one of the most serious and life-threating health problem worldwide [1]. To our knowledge, HBV or hepatitis C virus (HCV) infections is the most leading cause of HCC [18]. As hepatitis B virus was prevalent in China, the cases in our study were almost hepatitis B related HCC. Although there are studies reveal that HBV accelerate HCC via multiple mechanisms, most of the important is that HCC usually developed in the presence of chronic liver diseases, cirrhosis, and associated with impaired liver function [19, 20]. As we known, the long-term survival of HCC patients greatly depends on the well-preserved liver function as well as early-stage HCC. Although at least there are 18 HCC staging systems now available, UICC/AJCC TNM staging system and BCLC staging system are both among the most common HCC classification and scoring systems [4]. UICC/AJCC stage T3 (stage IIIa/IIIb) HCC patients, which were considered as intermediate or advanced stage in BCLC system, remains even extremely poor in prognosis. Especially as for stage IIIb cases, portal vein thrombosis develops extremely high portal hypertension which at last results in life-threatening bleeding esophageal and/or gastric varices, liver dysfunction, intrahepatic dissemination of HCC and/or distant metastasis.

The outcomes of treatments for those patients with such advanced stage have been disappointing in a long time. Curative options such as hepatic resection (HR), liver transplantation (LT) or radiofrequency ablation (RFA) were not recommended in Europe and North America. Although TACE might offer improved overall survival benefits in some non-randomized control trials, it is not yet recommended by practice guidelines [21–23]. On the other hand, therapy choices for those patients in such stage in Asian-Pacific areas may be pretty different. Surgical resection was recognized as the last but not least option for these patients to obtain long-term survival [2, 3, 5, 6]. Several studies have reported that radical resection of the tumor and involved vessels can prolong survival and may eventually offer a chance of cure in selected cases [3, 12, 24, 25]. However, even in these areas, there is still controversy over optimum treatment strategy for HCC patients in these stage, regardless guidelines for practice. Although there were several studies comparing en bloc with peeling off technique in the resection for HCC with portal vein tumor thrombus (PVTT), we conducted one of the largest study population and longest follow-up data in our previous study that demonstrated en bloc HR yielded more preferable survival outcomes over peeling off resection for HCC with PVTT [3, 26, 27]. In this study, we demonstrated that hepatic resection contributed to better OS compared with TACE in UICC/AJCC stage IIIa/IIIb HCC cases. The medium OS in HR group were 17.8 m, which were 6 months longer than that of TACE group (11.8 m). The 1, 3, and 5-year OS in HR group was significantly higher than that of TACE group, respectively, (log rank = 19.908, *P* < 0.01). Several studies reported that the response rate to TACE was around 40% with supra-selective technique, and the OS of the patients after TACE treatment ranged from 16 months to 25 months and even 48 months in selective recent series [28, 29]. However, the response rate to TACE was about 25% in this study. The median OS was 11 months (12 months after matching), which was consistent to the previous findings [15, 30, 31]. One of the reasons might be that the clinical stage of the included cases in this study was UICC stage T3 HCC. The mean size of tumor was 9.5 cm, with more than one lesion in most cases, or with portal vein involved. Kadalayil, et al. [32] has reported a simple prognostic scoring system, the Hepatoma arterial-embolization prognostic (HAP) score. In this prognostic scoring system, patients with low albumin (< 36 g/dl), high bilirubin (> 17 μmol/l) or α-fetoprotein (AFP) (> 400 ng/ml), and large tumor size (> 7 cm) were associated with increased risks of death when underwent TACE. In this study, the HAP score was a little bit high (albumin, 41.1 g/dl; bilirubin 17.8 μmol/l; tumor size, 8.6 cm; and 47% of cases with AFP > 400 ng/ml). However, the clinical and pathologic data in this study was consistent well with our previous studies [3, 12, 14]. According to the guideline of diagnosis and treatment of hepatocellular carcinoma of China [33], the cases with UICC T3 HCC were suitable for TACE treatment. Although before matching, more than 50% of the patients received only one TACE session, and 50% of these patients had disease progression after the session, the medium OS in TACE group was 10.9 (95% CI, 9.7–12.1) months, which was consistent with other studies [34, 35].

The OS in HR group was lower than those reported in other researches [36, 37]. However, the results in this study were consistent with those we previously reported [3, 12, 14]. One of the reasons might be that the patients enrolled in our studies were at a more advanced stage. Some patients with advanced HCC might benefit from resection [38, 39]. In this study, 36% of the patients after matching had one tumor, the most frequent liver disease etiology was HBV infection, the median age of the patients was 49.5 y, and the platelet count was 200 × 10^9^/L (which means no portal hypertension). In view of these characteristics, a surgical management should be done.

Until now, there have been several studies and meta-analysis accessing HR and TACE in the management of intermediate or advanced stage of HCC [25, 40–44]. However, to our knowledge, the study we presented here was one of the several studies to access the survival outcome of HR versus TACE in UICC/AJCC stage IIIa/IIIb HCC patients [12, 45, 46]. Moreover, this study comprised the largest study population and presented the longest follow-up data reported to date [3, 12, 25–37]. At last but not least, our findings were obtained after PSM which balanced patient demographics, liver functions, and tumor characteristics between two groups. Therefore, it provided us the most important data that might be used to establish an optimal strategy for the management of UICC stage IIIa/IIIb HCC patients.

In terms of safety, our study revealed that either HR or TACE was generally well tolerated and just several manageable adverse events occurred in patients with UICC stage T3 HCC patients. Although patients in TACE group were less likely to develop grades 3–4 edema, ascites, and pleural effusion before matching, patients in HR group were more likely to develop pleural effusion after matching. These were similar to those results reported in the previous studies [3, 12, 25, 45–48].

In this study, we performed univariate and multivariate analysis to examine demographics and clinical characteristics associated with prognosis. Although Cox analysis showed that PT, tumor size, tumor numbers, UICC stage were independent prognostic factors, the hazard ratio was just a little scale, which seemed to be not so clear advantage for either arm. On the other hand, initial treatment of hepatic resection yielded a hazard ratio of 0.646 over TACE, which meant there was a 35.6% reduction in risk of death in HR group, that was a clear advantage in HR arm. Although Kadalayil, et al. [32] reported that α-fetoprotein (AFP) (> 400 ng/ml) was associated with increased risks of death when underwent TACE, in this study, the AFP level was not an independent prognostic variable. Other studies suggested some risk factors for OS in UICC stage T3 HCC, such as ALB < 3.5 g/dL, tumor size more than 55 mm, multiple tumors, peeling off thrombectomy in HR, and treatment option of TACE alone, et al [3, 14, 46, 47] These observations were partly compatible with our current results. Not surprisingly, patients with long-term OS were more likely to have normal PT time, smaller tumors, and less likely to be multiple tumors.

Due to retrospective study, our study ineluctably had some limitations. The most significant one was lack of a well-balanced randomization. The treatment choices were recommended by our Multiple Disciplinary Team (MDT) in consideration of various clinical features and guidelines available, which were more likely to increase the possibility of unbalanced treatment allocation through the treatment distribution and potential selection bias occurred. Although some studies revealed that propensity scores matching (PSM) methods was not necessarily superior to conventional covariate adjustment, it was still an increasingly popular method to balance bias in observational studies [49]. Therefore, the problem of imbalance was supposed to be partially addressed by using propensity score matching that yielded similar baseline characteristics between two groups. Among the risk factors of OS, an additional analysis to define a subgroup which is really saved by HR compared to TACE in even UICC T3 HCC would give more practical information for the treat. However, we did not perform the subgroup analysis. This is the second limitation of this study.

## Conclusions

Our study revealed that TACE was an option for UICC stage T3 HCC patients. However, potentially radical hepatic resection (HR) yielded a result of overall survival advantage on TACE for UICC stage T3 HCC patients. Therefore, HR seemed to represent the optimal therapy strategy for the management of UICC stage T3 HCC and should be recommended as a preferable treatment especially for patients with a good underlying liver function.

## Abbreviations

AASLD: the American Association for the Study of Liver Diseases
AEs: adverse events
AFP: alpha-fetoprotein
AJCC: the American Joint Committee on Cancer
ALBI: albumin–bilirubin
AST: aspartate aminotransferase
BCLC: Barcelona clinic liver cancer
CI: confidence interval
CT: Computer tomography
HAP: Hepatoma arterial-embolization prognostic
HBV: hepatitis B virus
HCC: hepatocellular carcinoma
HCV: hepatitis C virus
HR: hepatic resection
LT: liver transplantation
MDT: Multiple Disciplinary Team
MRI: magnetic resonance imaging
NCI: the National Cancer Institute
OS: overall survival
POH: postoperative hemorrhage
PS: performance status
PSM: propensity score matching
PT: prothrombin time (PT)
PVTT: portal vein tumor thrombus
RFA: radiofrequency ablation
TACE: transarterial chemoembolization
TBL: total bilirubin
TNM: tumor-node-metastasis
TRD: treatment-related deaths
TTP: time to progression
UGIH: upper gastrointestinal hemorrhage
UICC: the Union for International Cancer Control

## References

[1] Ferlay J, Soerjomataram II, Dikshit R (2015). Cancer incidence and mortality worldwide: sources, methods and major patterns in GLOBOCAN 2012. Int J Cancer.

[2] Bruix J, Reig M, Sherman M (2016). Evidence-based diagnosis, staging, and treatment of patients with hepatocellular carcinoma. Gastroenterology.

[3] Zhang YF, Le Y, Wei W (2016). Optimal surgical strategy for hepatocellular carcinoma with portal vein tumor thrombus: a propensity score analysis. Oncotarget.

[4] Faria SC, Szklaruk J, Kaseb AO (2014). TNM/Okuka/Barcelona/UNOS/CLIP international multidisciplinary classification of hepatocellular carcinoma: concepts, perspectives, and radiologic implications. Abdom Imaging.

[5] Bruix J, Sherman M (2011). Management of hepatocellular carcinoma: an update. Hepatology.

[6] Omata M, Cheng AL, Kokudo N (2017). Asia-Pacific clinical practice guidelines on the management of hepatocellular carcinoma: a 2017 update. Hepatol Int.

[7] Sangiovanni A, Colombo M (2016). Treatment of hepatocellular carcinoma beyond international guidelines. Liver Int.

[8] Ishizawa T, Hasegawa K, Aoki T (2008). Neither multiple tumors nor portal hypertension are surgical contraindications for hepatocellular carcinoma. Gastroenterology.

[9] Cucchetti A, Ercolani G, Vivarelli M (2009). Is portal hypertension a contraindication to hepatic resection?. Ann Surg.

[10] D’Agostino RB (1998). Propensity score methods for bias reduction in the comparison of a treatment to a non-randomized control group. Stat Med.

[11] Loux TM (2015). Randomization, matching, and propensity scores in the design and analysis of experimental studies with measured baseline covariates. Stat Med.

[12] Zhong C, Guo RP, Li JQ (2009). A randomized controlled trial of hepatectomy with adjuvant transcatheter arterial chemoembolization versus hepatectomy alone for stage III a hepatocellular carcinoma. J Cancer Res Clin Oncol.

[13] Zhang YF, Guo RP, Zou RH (2016). Efficacy and safety of preoperative chemoembolization for resectable hepatocellular carcinoma with portal vein invasion: a prospective comparative study. Eur Radiol.

[14] Zhang YF, Zhou J, Wei W (2016). Intermediate-stage hepatocellular carcinoma treated with hepatic resection: the NSP score as an aid to decision-making. Br J Cancer.

[15] Zhang YF, Wei W, Wang JH (2016). Transarterial chemoembolization combined with sorafenib for the treatment of hepatocellular carcinoma with hepatic vein tumor thrombus. Onco Targets Ther.

[16] Institute NC (2006). Common terminology criteria for adverse events v3. 0 (CTCAE). Cancer.

[17] Johnson PJ, Berhane S, Kagebayashi C (2015). Assessment of liver function in patients with hepatocellular carcinoma: a new evidence-based approach-the ALBI grade. J Clin Oncol.

[18] Parkin DM (2006). The global health burden of infection-associated cancers in the year 2002. Int J Cancer.

[19] Chen CJ, Yang HI, Su J (2006). Risk of hepatocellular carcinoma across a biological gradient of serum hepatitis B virus DNA level. JAMA.

[20] Wang M, Xi D, Ning Q (2017). Virus-induced hepatocellular carcinoma with special emphasis on HBV. Hepatol Int.

[21] Chung GE, Lee JH, Kim HY (2011). Transarterial chemoembolization can be safely performed in patients with hepatocellular carcinoma invading the main portal vein and may improve the overall survival. Radiology.

[22] Xue TC, Xie XY, Zhang L (2013). Transarterial chemoembolization for hepatocellular carcinoma with portal vein tumor thrombus: a meta-analysis. BMC Gastroenterol.

[23] Kishore S, Friedman T, Madoff DC (2017). Update on embolization therapies for hepatocellular carcinoma. Curr Oncol Rep.

[24] Roayaie S, Jibara G, Taouli B (2013). Resection of hepatocellular carcinoma with macroscopic vascular invasion. Ann Surg Oncol.

[25] Kamiyama T, Orimo T, Wakayama K (2017). Survival outcomes of hepatectomy for stage B hepatocellular carcinoma in the BCLC classification. World J Surg Oncol.

[26] Inoue Y, Hasegawa K, Ishizawa T (2009). Is there any difference in survival according to the portal tumor thrombectomy method in patients with hepatocellular carcinoma?. Surgery.

[27] Chok KS, Cheung TT, Chan SC (2014). Surgical outcomes in hepatocellular carcinoma patients with portal vein tumor thrombosis. World J Surg.

[28] Sieghart W, Hucke F, Pinter M (2013). The ART of decision making: retreatment with transarterial chemoembolization in patients with hepatocellular carcinoma. Hepatology.

[29] Burrel M, Reig M, Forner A (2012). Survival of patients with hepatocellular carcinoma treated by transarterial chemoembolisation (TACE) using drug eluting beads. Implications for clinical practice and trial design. J Hepatol.

[30] Kim HC, Lee JH, Chung JW (2013). Transarterial chemoembolization with additional cisplatin infusion for hepatocellular carcinoma invading the hepatic vein. J Vasc Interv Radiol.

[31] Chung SM, Yoon CJ, Lee SS (2014). Treatment outcomes of transcatheter arterial chemoembolization for hepatocellular carcinoma that invades hepatic vein or inferior vena cava. Cardiovasc Intervent Radiol.

[32] Kadalayil L, Benini R, Pallan L (2013). A simple prognostic scoring system for patients receiving transarterial embolisation for hepatocellular cancer. Ann Oncol.

[33] Xie DY, Ren ZG, Zhou J (2017). Critical appraisal of Chinese 2017 guideline on the management of hepatocellular carcinoma. Hepatobiliary Surg Nutr.

[34] Luo J, Guo RP, Lai EC (2011). Transarterial chemoembolization for unresectable hepatocellular carcinoma with portal vein tumor thrombosis: a prospective comparative study. Ann Surg Oncol.

[35] Gorodetski B, Chapiro J, Schernthaner R (2017). Advanced-stage hepatocellular carcinoma with portal vein thrombosis: conventional versus drug-eluting beads transcatheter arterial chemoembolization. Eur Radiol.

[36] Hsu CY, Hsia CY, Huang YH (2012). Comparison of surgical resection and transarterial chemoembolization for hepatocellular carcinoma beyond the Milan criteria: a propensity score analysis. Ann Surg Oncol.

[37] Liu PH, Lee YH, Hsia CY (2014). Surgical resection versus transarterial chemoembolization for hepatocellular carcinoma with portal vein tumor thrombosis: a propensity score analysis. Ann Surg Oncol.

[38] Peng ZW, Guo RP, Zhang YJ (2012). Hepatic resection versus transcatheter arterial chemoembolization for the treatment of hepatocellular carcinoma with portal vein tumor thrombus. Cancer.

[39] Torzilli G, Donadon M, Belghiti J (2016). Predicting individual survival after hepatectomy for hepatocellular carcinoma: a novel nomogram from the “HCC East & West Study Group”. J Gastrointest Surg.

[40] Li Q, Wang J, Sun Y (2006). Efficacy of postoperative transarterial chemoembolization and portal vein chemotherapy for patients with hepatocellular carcinoma complicated by portal vein tumor thrombosis-a randomized study. World J Surg.

[41] Peng BG, He Q, Li JP (2009). Adjuvant transcatheter arterial chemo- embolization improves efficacy of hepatectomy for patients with hepatocellular carcinoma and portal vein tumor thrombus. Am J Surg.

[42] Yin L, Li H, Li AJ (2014). Partial hepatectomy vs. transcatheter arterial chemoembolization for resectable multiple hepatocellular carcinoma beyond Milan criteria: a RCT. J Hepatol.

[43] Qi X, Wang D, Su C (2015). Hepatic resection versus transarterial chemoembolization for the initial treatment of hepatocellular carcinoma: a systematic review and meta-analysis. Oncotarget.

[44] Li K, Wang HT, He YK (2017). New idea for treatment strategies for Barcelona clinic liver Cancer stages based on a network meta-analysis. Medicine (Baltimore).

[45] Li B, Yu J, Wang L (2003). Study of local three-dimensional conformal radiotherapy combined with transcatheter arterial chemoembolization for patients with stage III hepatocellular carcinoma. Am J Clin Oncol.

[46] Ochiai T, Ishii H, Yamamoto Y (2015). Significance of hepatectomy for AJCC/UICC T3 hepatocellular carcinoma. Anticancer Res.

[47] Zaydfudim VM, Vachharajani N, Klintmalm GB (2016). Liver resection and transplantation for patients with hepatocellular carcinoma beyond Milan criteria. Ann Surg.

[48] Okamura Y, Sugiura T, Ito T (2017). The optimal cut-off value of the preoperative prognostic nutritional index for the survival differs according to the TNM stage in hepatocellular carcinoma. Surg Today.

[49] Elze MC, Gregson J, Baber U (2017). Comparison of propensity score methods and covariate adjustment: evaluation in 4 cardiovascular studies. J Am Coll Cardiol.

